# The Tourette International Collaborative Genetics (TIC Genetics) study, finding the genes causing Tourette syndrome: objectives and methods

**DOI:** 10.1007/s00787-014-0543-x

**Published:** 2014-04-26

**Authors:** Andrea Dietrich, Thomas V. Fernandez, Robert A. King, Matthew W. State, Jay A. Tischfield, Pieter J. Hoekstra, Gary A. Heiman, the TIC Genetics Collaborative Group

**Affiliations:** 1 Department of Psychiatry, University Medical Center Groningen, University of Groningen, Groningen, The Netherlands; 2 Yale Child Study Center and Department of Psychiatry, Yale University School of Medicine, New Haven, CT USA; 3 Department of Psychiatry, University of California, San Francisco, USA; 4 Department of Genetics, The Human Genetics Institute of New Jersey, Rutgers, the State University of New Jersey, Life Science Building, 145 Bevier Road, Piscataway, NJ 08854-8082 USA

**Keywords:** Genes, Methods, Multiplex families, Rare variants, Repository, Tourette syndrome, Trios

## Abstract

Tourette syndrome (TS) is a neuropsychiatric disorder characterized by recurrent motor and vocal tics, often accompanied by obsessive–compulsive disorder and/or attention-deficit/hyperactivity disorder. While the evidence for a genetic contribution is strong, its exact nature has yet to be clarified fully. There is now mounting evidence that the genetic risks for TS include both common and rare variants and may involve complex multigenic inheritance or, in rare cases, a single major gene. Based on recent progress in many other common disorders with apparently similar genetic architectures, it is clear that large patient cohorts and open-access repositories will be essential to further advance the field. To that end, the large multicenter Tourette International Collaborative Genetics (TIC Genetics) study was established. The goal of the TIC Genetics study is to undertake a comprehensive gene discovery effort, focusing both on familial genetic variants with large effects within multiply affected pedigrees and on de novo mutations ascertained through the analysis of apparently simplex parent–child trios with non-familial tics. The clinical data and biomaterials (DNA, transformed cell lines, RNA) are part of a sharing repository located within the National Institute for Mental Health Center for Collaborative Genomics Research on Mental Disorders, USA, and will be made available to the broad scientific community. This resource will ultimately facilitate better understanding of the pathophysiology of TS and related disorders and the development of novel therapies. Here, we describe the objectives and methods of the TIC Genetics study as a reference for future studies from our group and to facilitate collaboration between genetics consortia in the field of TS.

## Introduction

Tourette syndrome (TS) is a neuropsychiatric developmental disorder characterized by rapid, recurrent, non-rhythmic movements (motor tics) and vocalizations (vocal tics) [1]. TS is etiologically closely linked to other chronic tic disorders, such as chronic motor or vocal tic disorder. The worldwide prevalence of TS is estimated to be between 0.3 and 1 % and onset is typically in early childhood [2, 3]. Many children and adults with TS also meet criteria for comorbid obsessive–compulsive disorder (OCD, 30–50 %) [4], attention-deficit/hyperactivity disorder (ADHD, about 40–60 %) [3, 5, 6], or other comorbidities [7]. Although both drug and behavior therapies can often at least partially alleviate tic and comorbid symptoms and improve quality of life [8–10], current medications frequently have unwanted side effects and a substantial number of patients do not show positive or only limited responses to either drug and/or behavioral interventions [8–12]. Although the majority of young patients experience a spontaneous improvement of tic severity by young adulthood [6], about 25 % of patients with TS continue to have moderate to severe tic symptoms into adulthood [4, 6] and many individuals continue to suffer from the negative social consequences, even after the tics have subsided [12–14]. At present, there are insufficient data to guide the development of specifically targeted therapies. Thus, there is a pressing need for a better understanding of the pathophysiology of TS and related disorders to develop novel treatments that target the underlying cause(s).

While family and twin studies have consistently pointed to a significant genetic contribution, the molecular basis of TS is not well understood and progress in gene discovery has been slow relative to other neuropsychiatric disorders [15]. Since early segregation studies had suggested single-gene autosomal dominant transmission in TS [16–18], first gene discovery efforts were directed at very large pedigrees. These early investigations focused either on traditional linkage analysis or mapping of chromosomal abnormalities [19, 20]. However, later segregation studies have suggested a more complex inheritance pattern involving multiple genes (for a review, see [21]) and the model of a uniform single dominant gene no longer holds. Moreover, environmental factors (such as pre- and perinatal events), gene×environment interactions, and likely genetic heterogeneity add to the complexity of unraveling the etiology of TS [22, 23].

Although various genes and chromosomal regions have been implicated in TS etiology, to date, no single risk gene or genetic variation has been definitively established as a uniform cause or contributing factor for TS (for a review, see [21, 23]). The growing appreciation of the involvement of multiple genetic variants in TS fuelled the application of large genome-wide association study (GWAS) designs. While the study of common variants based on a case–control design in very large samples appears promising, in the only large GWAS study published to date, no locus reached the threshold for genome-wide significance [24].

Meanwhile, the burgeoning development of genomic technologies, including micro-arrays and next-generation sequencing, has fostered renewed interest in studying multiply affected family pedigrees to search for rare variants of large effects, variants that would not be captured in GWAS studies, which focus on common single-nucleotide polymorphisms (SNPs) of small effects [15, 25, 26]. Micro-arrays have also been used for studying de novo copy number variations (CNVs) by using parent–child trios; identifying de novo mutations possibly underlying TS can be enhanced by investigating non-familial forms of TS through the analysis of apparently simplex trios (i.e., an affected individual with both parents unaffected) [15, 21, 25, 26]. The studies of rare variants have yielded intriguing findings that implicate genes involved in histaminergic and serotonergic pathways ([27–29], see also [30]) and genes involved in promoting dendritic growth [20, 31].

To summarize, it is now recognized that TS is almost certainly the result of complex and heterogeneous inheritance involving both common and rare variants [21], which are likely interacting with environmental factors [22, 23]. Based on the rapid progress in other neurodevelopmental disorders such as autism and schizophrenia [32], it has become clear that large patient cohorts are essential for gene discovery efforts [15]. Large open-access repositories that include well-characterized probands and family pedigrees will promote innovative studies utilizing emerging technologies to understand the genetic architecture of complex diseases, including TS and related disorders [21, 23].

### The TIC Genetics study

The Tourette International Collaborative Genetics (TIC Genetics; http://tic-genetics.org) study was established in 2011 to further our understanding of the genetic architecture of tic disorders by developing a large sample of genotypically and phenotypically well-characterized affected probands and relatives. The study employs state-of-the-art genetic technologies to identify major genetic variants contributing to TS and the most commonly comorbid disorders, including OCD and ADHD. Specifically, TIC Genetics searches for likely deleterious mutations that are shared by affected individuals within multiply affected families. A second focus is searching for de novo mutations, particularly rare loss of function variants that occur in the same gene in more than one individual. Identifying de novo mutations is an especially promising gene-finding approach, given the expected higher signal-to-noise ratio when comparing the affected child versus unaffected parents, using CNV, whole-exome and whole-genome analysis [33]. We also aim to explore interactions between susceptibility genes and perinatal environmental factors with regard to the clinical phenotype and will investigate genome-wide gene expression of peripheral blood mRNA from TS probands carrying selected CNVs of interest or segregating rare mutations. We will evaluate the contribution of the entire allelic spectrum to TS.

The TIC Genetics study is an international collaboration of more than twenty sites from the USA, Europe, and South Korea, including academic research centers and mental health care settings led by experienced clinicians and investigators in TS and related disorders. The study sites recruit and clinically assess individuals with TS and their family members, and collect blood samples for DNA and RNA extraction. The clinical and biomaterial data are part of a sharing repository of the National Institute for Mental Health (NIMH) Center for Collaborative Genomics Research on Mental Disorders in the USA and will be made available to the research community at large to hasten the identification of causal genetic factors and facilitate better understanding and treatment of this often impairing disorder. The repository includes DNA, transformed cell lines, and RNA. Hereafter, we describe the methods of the TIC Genetics study and how to gain access to the repository.

## Methods

### Subjects

Recruitment started in September 2011 and is ongoing. The TIC Genetics study’s current aim is to ascertain at least 1,548 subjects from multiplex families (affected family pedigrees) or parent–child trios (a trio includes an affected proband with both biological parents). Probands must meet criteria for TS or another chronic tic disorder (see Table 1 for definitions). Multiplex families consist of an affected proband with a tic disorder and at least two other relatives (up to the fourth degree) with a tic disorder or OCD (at least one of the relatives must have a tic disorder, others may also have OCD). In those families, unaffected first-degree relatives of affected participants are also recruited, as well as connecting relatives (e.g., grandparents of first cousins). Enrollment of bilineal pedigrees (i.e., tics in both paternal and maternal sides) is permitted, but discouraged as they are less informative [34]. Apart from multiplex families, we focus on recruiting simplex trios in which only the proband but neither parent nor siblings (and preferably no other known family member) has a tic disorder.

**Table 1:** Overview of measures. Clinical assessments and diagnoses are based on self- or parent-on-child reports and a subsequent clinical interview in accordance with Diagnostic and Statistical Manual of Mental Disorders (DSM)-IV-TR criteria [35]

Topics	Measures	Measurement instruments, examples of variables
Demographic information and health indicators		Tourette Syndrome Association Genetic Linkage Consortium’s Family Self-Report Questionnaire (TSA, January 1995; http://www.findTSgenes.org)
	Demographics	Race, ethnicity, schooling, parent education/occupation, birth order, handedness, etc.
	Subject’s medical history	Range of pulmonary, dermatologic, allergic, cardiovascular diseases, neurological conditions, congenital anomalies, genetic syndromes, etc.
	Psychotropic medication use (lifetime and past two weeks)	Broad range of neuroleptics, selective serotonin reuptake inhibitors, other antidepressants, mood stabilizers, benzodiazepines, stimulants etc.
	Family history of tic and other relevant psychiatric and medical disorders	OCD, ADHD, hair pulling, autism spectrum disorders, mental retardation, neurological disorders, genetic syndromes, etc., of family members
Psychopathological disorders and symptoms, lifetime and past week		
Tic disorders	Tourette syndrome	Yale Global Tic Severity Scale (YGTSS, Leckman et al. [36])
	Chronic tic disorders (chronic motor or vocal tic disorder, or combined subtype^a^)	
	Transient tic disorder	
	Provisional tic disorder (DSM-5 [47])	
	Tic disorder-NOS	
Obsessive–compulsive disorder (OCD) or OC symptoms	OCD Subclinical OCD OC symptoms	Yale–Brown Obsessive–Compulsive Scale (Y-BOCS, Goodman et al. [38, 39])
Trichotillomania		Questions on past and present hair pulling, pulling eye-lashes or eyebrows resulting in noticeable hair loss
Attention-deficit/hyperactivity disorder (ADHD)	Combined type Predominantly inattentive type Predominantly hyperactive–impulsive type Subclinical ADHD	Swanson Nolan and Pelham-IV (SNAP-IV, Swanson et al. 1992) [43]
Other medical or psychiatric history, or aggressive episodes		Neurological, medical, or genetic disorders; autism spectrum disorders, psychotic disorders, anxiety disorders, mood disorders, externalizing disorders, etc., by clinician review
Environmental risk factors	Prenatal, perinatal, and developmental history	Pregnancy, Birth, and Development Questionnaire (Modified schedule for risk and protective factors early in development; Walkup and Leckman [51, 52]) Pregnancy (maternal age, paternal age, pregnancy duration, special medical procedures or problems during pregnancy, medication use, use of substances [smoking, alcohol, street drugs, caffeine]) Labor and delivery (birth weight, gestational age, complications, multiple pregnancy, medications, premature birth) Newborn period (APGAR scores, medical concerns, problems, and interventions) First years of life (developmental milestones)
Biomaterials	DNA, transformed cell lines, RNA	

^a^Separate category not covered in the DSM for individuals with a history of only a single motor tic and at least one vocal tic, with onset by age 18 years

Subjects are not excluded based on age, gender, race, or ethnicity. Exclusion criteria are limited to inability to obtain appropriate informed consent, refusal of blood sampling by minors, or situations in which, in the physician’s best judgment, it would not be in the subject’s best interest to be enrolled.

Subjects are recruited from all collaborating sites based on new referrals, previously diagnosed tic disorder patients, and occasionally via patient organizations. Each clinical recruiting center has obtained separate study approval from their local Human Investigations Committee. Adults, as well as the child’s biological parent(s) (or legal guardian) in case of minors, provide written informed consent and the participating child gives written or oral assent before entering the study. The informed consent specifies inclusion into a sharing repository through the NIMH Center for Collaborative Genomics Research on Mental Disorders, USA (www.nimhgenetics.org) as a requirement. All participating subjects give consent for submission of anonymized data to, and distribution from, an NIMH repository.

### Data collection procedure

**Fig. 1:**
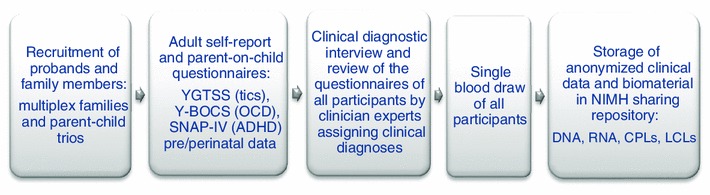
Data collection procedure. *YGTSS* Yale Global Tic Severity Scale, *Y-BOCS* Yale–Brown Obsessive–Compulsive Scale, *OCD* obsessive–compulsive disorder, *SNAP-IV* Swanson Nolan and Pelham-IV ADHD rating scale, *ADHD* attention-deficit/hyperactivity disorder, *NIMH* National Institute of Mental Health, *CPLs* cryopreserved lymphocytes, *LCLs* lymphoblastoid cell lines

All collaborating centers follow the same standardized data collection procedures (Fig. 1). Consenting probands and their family members are invited for a single visit to the respective centers, providing a set of completed standardized and well-validated tic, OCD, and ADHD rating instruments (adult self-report and/or parent-on-child report). Experienced clinicians specialized in TS review the instruments during an in-person semi-structured clinical interview of all participants regarding the presence or absence of lifetime and current tic and other neuropsychiatric disorders and assign clinical diagnoses. After the evaluation, the coded de-identified demographic data and clinical diagnoses are entered into our online password-protected data-entry system. Here, the clinician rates each diagnostic criterion for tic disorder, OCD, and ADHD and the corresponding disorder as “present”, “absent”, or “unable to rate” based on the Diagnostic and Statistical Manual of Mental Disorders—Fourth edition, Text Revision (DSM-IV-TR) [35]. The online system utilizes internal algorithms to ensure complete data entry and the appropriate match between the reported diagnostic criterial symptoms and the assigned corresponding diagnoses for each of these disorders. The evaluating clinician also provides a short diagnostic narrative report for each subject. Scans of the completed de-identified questionnaires are sent to the data coordination center at Rutgers University. Finally, blood of participants (total of 30 ml) is collected by a single venipuncture and immediately shipped to the NIMH Center for Collaborative Genomics Research on Mental Disorders at RUCDR (RUCDR Infinite Biologics, http://www.rucdr.org) at Rutgers University, Piscataway, NJ, USA.

### Clinical assessments

#### Adult self-report and parent-on-child report questionnaires followed by clinician review

Table 1 shows an overview of the clinical assessment measures. Adult self-reports and parallel parent-on-child report questionnaires are used to assess subjects’ demographics, medical history, psychopathology, and selected environmental factors, as well as family members’ psychiatric history.

Lifetime and current (i.e., past week) tic, obsessive–compulsive, trichotillomania (a condition, sometimes associated with TS), and ADHD symptoms corresponding to DSM-IV-TR [35] criteria are initially assessed by adult self- and parallel parent-on-child ratings which are reviewed during a follow-up clinical interview by board-certified clinicians who are experienced in the evaluation and treatment of TS and who assign the clinical diagnoses based on that review. Included are the widely used, highly reliable and well-validated Yale Global Tic Severity Scale (YGTSS) [36, 37] and Yale–Brown Obsessive–Compulsive Scale (Y-BOCS) [38–40]. While the latter were originally developed as semi-structured interviews, self-report and parent-on-child questionnaires for each have been developed and used (see in [41, 42]). TIC Genetics uses modified versions of these self- or parent-on-child reports to provide a standardized initial symptom inventory, followed by clinician review and validation of symptom reports (see [41] and [42] for a comparable procedure). The original YGTSS contains a tic section consisting of an inventory of the various lifetime and current motor and vocal tic types and a severity section, rating the number, frequency, intensity, complexity, and interference due to tics on a six-point Likert scale for motor and vocal tics separately, and an overall impairment scale. The modified YGTSS version used in TIC Genetics has left out the number and complexity of tics as well as the assessment of impairment, as these are not strictly needed to establish a tic disorder diagnosis. The Y-BOCS comprises an inventory of 91 types of obsessions and compulsions across various domains followed by ratings of time spent, interference, distress, resistance, and level of control, separately for obsessions and compulsions. All original Y-BOCS sections are used in TIC Genetics. Finally, the reliable and well-validated Swanson Nolan and Pelham-IV ADHD (SNAP-IV) rating scale [43–45] is used to assess attention-deficit/hyperactivity symptoms based on self- or parent-on-child reports of symptoms and impairment (due to specific ADHD symptoms) present during the primary school years when not on medication for ADHD. The SNAP-IV is a widely used tool for the assessment of ADHD subtypes, scoring each nine items on inattention and hyperactivity–impulsivity symptoms during childhood on a four-point Likert scale ranging from “not at all” to “very much”; additional items assess age of onset and degree and pervasiveness of any impairment.

The questionnaires have been translated from English into Danish, Dutch, German, Korean, and Spanish by respective native speakers well-experienced with clinical diagnostic instruments and were largely based on pre-existing translations circulating in the different countries. Additional back-translations into English were done by an independent colleague to assure the high quality of the translations.

The set of TIC Genetics assessments described here is also used in other large genetic cohorts studies of TS (e.g., Tourette Syndrome Association International Consortium for Genetics, TSAICG, www.findTSgenes.org, [41, 42], TSA Genetic Linkage Consortium’s Family Self-Report Questionnaire, January, 1995; European Multicenter Tics in Children Studies, www.emtics.eu), thereby facilitating future replication studies and meta-analyses.

#### Clinical diagnoses

As presented in Table 1, the clinical information collected from the self-report and parent-on-child report questionnaires which are validated by clinician interview are used to assess the lifetime and current (i.e., past week) presence or absence of a tic disorder, OCD, trichotillomania, and ADHD, in accordance with strict DSM-IV-TR criteria [35]. The presence or absence of sub-syndromal symptom levels of OCD or ADHD is also rated, using the TSAICG conventions [46]. The assigned diagnostic categories of tic disorders also include provisional tic disorder based on the DSM-5 [47] as well as a separate category not covered in the DSM, chronic tic disorder combined subtype, for individuals who have a verified history, with onset by age 18 years, of only a single motor tic and at least one vocal tic. When there is diagnostic uncertainty (e.g., poor informant, uncertain historical data in an adult subject), clinicians can opt for an “unable to rate” diagnosis to reduce false negative diagnoses.

Because TIC Genetics’ priority is to acquire as large a sample of TS probands and relatives despite the constraints of limited funding, we have focused on the detailed assessment of TS-related symptoms and the most common comorbidities of OCD, ADHD, and trichotillomania. As a result, we were not able to deploy the resources to systematically query or validate the presence of other forms of psychopathology, either by interview or broad-based symptom checklists. However, based on the subjects’ (or parents’) responses on the medical history and psychotropic medication portion of the questionnaire, clinicians queried and reviewed with the informant any endorsed indication of past or present treatment for conditions such as autism spectrum disorder, anxiety or mood problems, and/or significant neurological, medical, or genetic disorders.

When the clinician judges that there is an atypical presentation of tic symptoms (e.g., onset after adolescence, potential stereotypies rather than true tics) or the presence of coexisting or potentially confounding psychiatric and medical conditions (e.g., autism spectrum disorder, psychosis, anxiety disorder, depression, and neurological or genetic syndromes [7]), a corresponding flag is entered into the database, to facilitate review. In addition, a short diagnostic narrative is written for each subject, briefly summarizing salient patient history and discussing possible atypical presentations and diagnostic uncertainties.

A Phenotype Assessment Sub-Committee, led by Drs. King and Heiman, conducts regular case reviews, either at random, on request, or focusing on cases flagged for atypical presentation, confounding other conditions, or in case of “unable to rate” diagnoses. In addition, the Sub-Committee maintains a continuous dialog with the clinicians and provides training materials and periodic training exercises with the goal of resolving any apparent ambiguities, arriving at a consensus diagnoses with the respective clinicians in ambiguous cases, ensuring consistent application of diagnostic criteria, and helping to maintain reliability and validity. Given the large scale of the study a formal best estimate diagnostic consensus process by independent expert reviewers on each subject appeared not to be feasible.

### Creation of permanent cell lines, whole-blood DNA, and transcriptomes

Each subject donates about 30 ml of blood (four tubes) and these are immediately shipped to the NIMH Center for Collaborative Genomics Research on Mental Disorders at RUCDR (RUCDR Infinite Biologics, http://www.rucdr.org) to be processed. One tube (ACD Solution A, 8.5 ml) is used to create cryopreserved lymphocytes (CPLs) that can later be converted to Epstein–Barr virus-transformed permanent lymphoblastoid cell lines (LCLs) or induced pluripotent stem cells (iPSCs). Two EDTA tubes (10 ml) are used to extract whole-blood DNA and RNA is extracted from one PAXgene™ tube (2.5 ml) for future transcriptomic studies.

### Data management and ethical issues

All anonymized clinical and biomaterial data from all participating centers are stored indefinitely in a central database within the NIMH Center for Collaborative Genomics Research on Mental Disorders at RUCDR (RUCDR Infinite Biologics, http://www.rucdr.org). The coded de-identified clinical diagnostic information is entered by each center into the online encrypted and password-protected diagnostic system via the TIC Genetics website (http://tic-genetics.org). Thus, no identifying personal information is sent from the participating sites or received by the data coordinating center or the repository. The subject’s identity is known only to the local research team of the collaborating centers, where the subject’s code (consisting of a unique site, family and subject ID number) is kept in a secured place for a certain period of time and subsequently anonymized in accordance with local medical-ethical regulations. The study is governed by the National Institutes of Health’s overall Human Investigations policies and reviewed and approved by each site’s Human Investigations Committee. The included subjects consent to the anonymized storage of biomaterials in the database. Finally, a publication committee regulates the group’s access to the database and publication plans.

### Sample description

Table 2 presents the current number of singleton probands, parent–child trios, and multiplex families in the repository. Table 3 shows the distribution of clinical diagnoses. The ongoing TIC Genetics repository currently (as of September 3rd, 2013) includes a total of 988 subjects (57 % males; 91.5 % white, 1.5 % black, 7.6 % Asian, and 3.3 % of mixed or unknown race) with available DNA and clinical evaluations; of these 492 (49.8 %) have TS or another chronic tic disorder. The repository consists of 274 (27.7 %) probands and 714 (72.3 %) relatives of whom 378 (52.9 %) are affected with a tic disorder and/or OCD, and 324 (45.4 %; 32.8 % of the whole sample) are unaffected with either tics or related disorders (i.e., absence of tic disorder, OCD, subclinical OCD, OC symptoms, trichotillomania, and ADHD).

**Table 2:** Number of singleton probands, parent–child trios, multiplex families, and other families in the repository (*N* = 988 subjects)

	Narrow model *N*	Intermediate model *N*	Broad model *N*
Singleton probands^a^	26	26	26
Trios^b^	218	218	218
* Simplex trios* ^c^	91	91	78
All Multiplex families^d^	53	62	76
Multiplex families with 3 affected	34	39	46
Multiplex families with 4 affected	12	14	20
Multiplex families with 5+ affected	7	9	10
Other families^e^	58	57	55

Data can be analyzed using different diagnostic models of family members’ affectedness: Narrow (affected with Tourette syndrome or another chronic tic disorder), Intermediate (affected with any type of tic disorder, including transient tic disorder, provisional tic disorder, and tic disorder-NOS), and Broad (affected with any type of tic disorder and/or obsessive–compulsive disorder)

^a^A proband is the index patient affected with Tourette syndrome or another chronic tic disorder

^b^Total number of parent–child trios, i.e., affected proband and both biological parents; these trios can be part of a multiplex family and relatives can be affected

^c^Without known relatives affected with a tic disorder (or obsessive–compulsive disorder as in the broad model; note that this has resulted in a lower number of simplex trios)

^d^Mean number of subjects per multiplex family [between *M* = 5.3 and *M* = 5.7, range 3–15] and mean number affected [*M* = 3.7, range 3–9, all models]

^e^Families with two affected persons, or ‘trios’ with a missing parent

**Table 3:** Frequency of clinical diagnoses per sex across probands, relatives, and the total sample (*N* = 988 subjects)

	*N* *M* (range) age yrs % sex	Tourette syndrome	Chronic tic disorder^a^	Other tic disorders^b^	OCD	Sub-clinical OC disorder and OC symptoms	Trichotillomania	ADHD^c^
		*N* (%) m/f	*N* (%) m/f	*N* (%) m/f	*N* (%) m/f	*N* (%) m/f	*N* (%) m/f	*N* (%) m/f
Probands^d^	274 15.9 (5–74) 77 % male	260 (94.9) 198/62	14 (5.1) 13/1	n/a	130 (47.4) 96/34	57 (20.8) 45/12	12 (4.4) 9/3	110 (36.5) 88/22
Relatives	714 40.9 (4–83) 49.3 % male	131 (18.3) 79/52	87 (12.2) 42/45	41 (5.7) 22/19	119 (16.7) 45/74	130 (18.2) 64/66	25 (3.5) 8/17	63 (8.8) 32/31
Total sample	988 28.4 (4–83) 57 % male	391 (39.6) 277/114	101 (10.2) 55/46	41 (4.2) 22/19	249 (25.2) 141/108	187 (18.9) 109/78	37 (3.7) 17/20	173 (17.5) 120/53

*OCD* obsessive–compulsive disorder, *OC* obsessive–compulsive, *ADHD* attention-deficit/hyperactivity disorder, *m* males, *f* females. Note that due to comorbidity across the diagnostic categories counts do not add up to the total. Of the 492 subjects with Tourette syndrome or chronic tic disorder, 23.6 % have comorbid OCD only, 14.2 % comorbid ADHD only, 16.9 % both comorbid OCD and ADHD, 3.9 % comorbid autism spectrum disorder, 3.5 % comorbid anxiety disorder, and 6.5 % comorbid mood disorder

^a^Motor or vocal tic disorder, or a separate combined subtype (defined by only a single motor tic and at least one vocal tic, with onset by age 18 years)

^b^Transient tic disorder, provisional tic disorder, and tic disorder-NOS

^c^Includes combined, predominantly inattentive, and predominantly hyperactive–impulsive type

^d^Index patient affected with Tourette syndrome or another chronic tic disorder

### Genetic data analyses

#### Multiplex families

Whole-exome and SNP genotyping are performed on multiplex families. Genotyping data are used to detect CNVs using multiple algorithms [28] and to perform an affected-only parametric linkage analysis. Linkage analysis will be performed using Illumina Omni BeadChip genotype data, pruned to a subset of well-spaced SNP probes, minimizing those in high linkage disequilibrium. Those families yielding the highest LOD scores (logarithm [base 10] of odds) will be identified. All rare coding variants that are plausibly disruptive (nonsense mutations including frameshift indels, missense mutations at highly conserved residues, and substitutions at canonical splice sites) and not present in existing control databases will be confirmed by Sanger sequencing and then evaluated for segregation in all affected family members. Further studies will be considered for segregating mutations for which more than one family shows rare segregating mutations in the same gene. Follow-up will include sequencing of the suspect gene in probands from every multiplex family, with further analysis of segregation in that pedigree if additional mutations are identified.

#### Simplex families (parent–child trios)

The exomes of affected subjects and their unaffected parents from simplex trios are sequenced using validated protocols for capture, sequence, analysis, and annotation [48]. For both inherited and de novo sequence variant detection, only positions with at least 8-fold coverage will be considered, based on 99.8 % (for inherited) and 96 % (for de novo) confirmation of variants at this threshold [48]. All de novo variants will be confirmed by re-amplification of native DNA and Sanger sequencing in all family members while blinded to affected status. Inherited variants will not be routinely validated due to the extremely high positive predictive value of such calls. The frequencies of sequence variants will be determined by comparison with the latest version of dbSNP (Single Nucleotide Polymorphism Database) and an existing collection of more than 2,000 unrelated whole-exome controls sequenced at Yale for other studies. A sequence variant will be classified as “novel” if it is not present in dbSNP and not present in the control exomes, “rare” if not novel and seen in <1 % of control exomes, or “common” if it is present in at least 1 % of control exomes. Variants will be mapped against the current RefSeq gene definitions (Reference Sequence database) to determine the effect of each variant on the resulting amino acid sequence. If multiple RefSeq gene isoforms are present, all will be manually examined for nonsense and splice site variants, as these are predicted to have the greatest impact on protein function. A variant will be considered to alter the splice site only if it disrupts the canonical two base-pair acceptor and donor sites adjacent to 98.5 % of exons in the human genome. These sites are among the most highly conserved base pairs in the human genome and such splice site variants have the potential to cause highly disruptive events, akin to nonsense variants and frameshift indels. Furthermore, variants will be mapped against a list of brain-expressed genes determined by expression array analysis across 57 postmortem brains and multiple brain regions in a recent study of the human brain transcriptome throughout development and adulthood [49].

#### Gene-environment

The TIC Genetics study also aims to systematically investigate the role of selected environmental determinants of tic disorders, including prenatal (e.g., exposure to alcohol, smoking or drug use), perinatal (e.g., delivery complications, birth weight and gestational age) and postnatal factors (e.g., infections) by (1) making comparisons between affected cases and controls by logistic regression; (2) relate prenatal, perinatal, and postnatal factors to tic severity and severity of comorbid conditions with linear regression within affected individuals; (3) identify critical time windows for the various post-natal environmental factors (exposure×time interactions); and (4) investigating gene×environment interactions, using a case–control design. A candidate gene approach will be chosen for the gene×environment studies, focusing on the replication of a comprehensive range of previously implied genes in TS by selection of SNPs that have been suggested in the available literature (e.g., [23, 24, 50]). The SNP selection covers all relevant genes in previously implicated catecholamine pathways (including dopamine, serotonin, histamine, GABA, and glutamate), non-neurotransmitter SNPs (e.g., SLITRK1, CNTNAP2), and SNPs implied by GWAS in TS, OCD, ADHD, and autism cohorts.

## Conclusion

The ongoing TIC Genetics study is an international collaborative effort aimed at understanding the genetic architecture of tic and related disorders. TIC Genetics has established an infrastructure to collect a large, well-characterized patient cohort in the service of identifying definitive risk genes for TS. The study’s database consists of detailed phenotypic data on TS and other tic disorders as well as commonly comorbid conditions (e.g., OCD, ADHD) along with DNA, transformed cell lines, and blood-derived RNA of affected individuals and their relatives, especially family pedigrees with multiply affected individuals and parent–child trios. The study promotes the use of novel cutting-edge gene-finding methods. Moreover, the collected anonymized data and biomaterial will be stored as a permanent resource for qualified researchers in the field of TS.

TIC Genetics study’s central mission is the creation of a sharing repository that will contribute to advancing the understanding of the genetics, biology, phenomenology, and treatment of TS. The clinical and biomaterial data are part of the NIMH Center for Collaborative Genomics Research on Mental Disorders, USA and will be made accessible by the NIMH within 6 months after the inclusion of the last subject to qualified scientists studying the genetics of TS and comorbid disorders. Information on how to gain access to clinical data, DNA, and transformed cell lines from NIMH can be found on https://www.nimhgenetics.org/access_data_biomaterial.php.

The TIC Genetics study has been designed to facilitate cooperation with other genetic TS consortia and researchers, with the promise to further advance the field of genetics of TS and related disorders. Ultimately, this may enhance our understanding of TS and further the development of novel therapies for patients with TS to increase their quality of life.

## Appendix

The TIC Genetics Collaborative Group members are Julia Bohnenpoll^a^, Lawrence W. Brown^b^, Keun-Ah Cheon^c^, Barbara J. Coffey^d,e^, Marta Correa^f^, Andrea Dietrich^g^, Stephanie Enghardt^h^, Thomas V. Fernandez^i^, Nikoline Frost^j^, Blanca Garcia-Delgar^k^, Donald L. Gilbert^l^, Dorothy E. Grice^d^, Julie Hagstroem^j^, Tammy Hedderly^m^, Gary A. Heiman^n^, Jeroen Heijmens Visser^o^, Pieter J. Hoekstra^g^, Isobel Heyman^p^, Hyun Ju Hong^q^, Chaim Huyser^r^, Robert A. King^i^, Young-Key Kim^s^, Young Shin Kim^i^, Yun-Joo Koh^t^, Sodahm Kook^u^, Samuel Kuperman^v^, Bennett Leventhal^e^, Andrea G. Ludolph^w^, Athanasios Maras^o^, Marcos Madruga-Garrido^x^, Pablo Mir^x,y^ Astrid Morer^z^, Tara Murphy^p^, Alexander Münchau^a^, Vivian op de Beek^r^, Kerstin J. Plessen^j^, Florianne Rademaker^g^, Veit Roessner^h^, Odette Schunke^a1^, Eun-Young Shin^c^, Dong-Ho Song^c^, Jungeun Song^s^, Matthew W. State^a2^, Jay A. Tischfield^n^, Jennifer Tübing^a^, Sina Wanderer^h^, Martin Woods^m^, Samuel H. Zinner^a3^

^a^Institute of Neurogenetics, University of Lübeck, Lübeck, Germany;

^b^Children’s Hospital of Philadelphia, Philadelphia, PA, USA;

^c^Yonsei University Severance Hospital, Seoul, South Korea;

^d^Icahn School of Medicine at Mount Sinai, New York, NY, USA;

^e^Nathan S. Kline Institute for Psychiatric Research, Orangeburg, NY, USA;

^f^Servicio Andaluz de Salud (SAS), Sevilla, Spain;

^g^University of Groningen, University Medical Center Groningen, Groningen, Department of Psychiatry, The Netherlands;

^h^Child and Adolescent Psychiatry, Faculty of Medicine of the Technical University Dresden, Dresden, Germany;

^i^Yale Child Study Center and Department of Psychiatry, Yale University School of Medicine, New Haven, CT, USA;

^j^Mental Health Services, Capital Region of Denmark and University of Copenhagen, Denmark;

^k^Fundació Clínic Recerca Biomèdica, Barcelona, Spain;

^l^Cincinnati Children’s Hospital Medical Center, Cincinnati, OH, USA;

^m^Evelina London Children’s Hospital GSTT, Kings Health Partners AHSC, London, UK;

^n^Rutgers, the State University of New Jersey, Department of Genetics and the Human Genetics Institute of New Jersey, Piscataway, NJ, USA;

^o^Yulius Academy and Division Child and Adolescent Psychiatry, Yulius Mental Health Organization, Rotterdam, The Netherlands;

^p^Great Ormond Street Hospital for Children, and Institute of Child Health, University College, London, UK;

^q^Hallym University Sacred Heart Hospital, Anyang, South Korea;

^r^De Bascule, Amsterdam, The Netherlands;

^s^National Health Insurance Service Ilsan Hospital, Goyang-shi, South Korea;

^t^Korea Institute for Children’s Social Development, Seoul, South Korea;

^u^MyongJi Hospital, Koyang, South Korea;

^v^University of Iowa Carver College of Medicine, Iowa City, IA USA;

^w^University of Ulm, Department of Child and Adolescent Psychiatry and Psychotherapy, Ulm, Germany;

^x^Instituto de Biomedicina de Sevilla (IBiS); and Hospital Universitario Virgen del Rocío/CSIC/Universidad de Sevilla, Spain;

^y^Centro de Investigación en Red sobre Enfermedades Neurodegenerativas (CIBERNED), Sevilla, Spain;

^z^Hospital Clinic; IDIBAPS; and CIBERSAM, Barcelona, Spain;

^a1^University Hospital Medical Center Hamburg-Eppendorf, Hamburg, Germany;

^a2^University of California, Department of Psychiatry, San Francisco, USA;

^a3^Seattle Children’s Hospital, Seattle, USA.
